# Experiences, Attitudes, and Practices of Resin Infiltration Use in Finland

**DOI:** 10.1155/ijod/3400651

**Published:** 2025-11-19

**Authors:** Päivi Havela, Jenni Kylkisalo, Hannu Vähänikkilä, Tarja Tanner, Vuokko Anttonen, Marja-Liisa Laitala

**Affiliations:** ^1^Research Unit of Population Health, Faculty of Medicine, University of Oulu, Oulu, Finland; ^2^Medical Research Center Oulu and Oulu University Hospital, Oulu, Finland; ^3^Northern Finland Birth Cohorts, Arctic Biobank, Infrastructure for Population Studies, Faculty of Medicine, University of Oulu, Oulu, Finland

**Keywords:** caries prevention, dental hygienist, mini-invasive, resin infiltration

## Abstract

**Objectives:**

The aim was to investigate the experiences, attitudes, and practices of resin infiltration (RI) among Finnish dentists.

**Methods:**

A Webropol-based questionnaire was sent via the Finnish Dental Association's regular newsletter to all members in April 2022. The questionnaire consisted of nine questions on experiences and opinions concerning RI with fixed options for answers. Additionally, methods that respondents had used for arresting initial caries lesions were asked with a possibility to choose several options. Descriptive analyses were performed using frequencies and proportions. For the analyses, respondents were grouped into RI users and nonusers. To analyze the differences between the groups, cross tabulation with Pearson's chi-squared test or Fisher's exact test were used.

**Results:**

Of the 416 respondents, 32.7% answered that RI is included in their selection of procedures. RI was considered an easy procedure by 46.7% of RI users and 19.9% of nonusers (*p* < 0.001). The scientific evidence of RI was reported to be promising significantly more often by RI users (41.5%) than nonusers (28.2%) (*p* < 0.001). Of the RI users, 48.1% thought that RI could be performed by dental hygienists, whereas 16.2% of nonusers thought so (*p* < 0.001). Of all respondents, approximate 90% instructed patients on better tooth brushing (*n* = 380), prescribed strong fluoride toothpaste (*n* = 363), or applied fluoride varnish/gel (*n* = 368) on the initial lesions for arresting initial caries lesions.

**Conclusions:**

The attitude of those who use RI in their clinical practice was more positive than that of nonusers. This indicates a need for further promotion of this minimally invasive approach to arresting and treating caries.

## 1. Introduction

According to the World Health Organization, oral diseases affect nearly 3.5 billion people, and untreated dental caries in permanent teeth is the most common noncommunicable disease according to the Global Burden of Diseases 2019 [1]. Controlling and preventing caries disease, as well as arresting the progression of active initial caries lesions, are keystones in the modern caries treatment schema [2]. Traditionally, active initial lesions on the occlusal fissures of molar teeth have been arrested with fissure sealants [3–5]. For arresting the progression of active initial interproximal lesions, especially those of an early stage, fluoride applications have been used [6, 7].

The resin infiltration (RI) technique was developed to provide a method specifically for arresting the progression of initial caries lesions in interproximal tooth surfaces (ICDAS scores 2 or 3) [2].^.^ In the RI method, a low viscosity resin penetrates the porous caries tissue by capillary forces. This technique aims to create a diffusion barrier with a low-viscosity resin inside the lesion to replace lost minerals [8]. After infiltration, the optic characteristics of the enamel become similar to those of the nonaffected enamel, and consequently, the tooth surface looks almost intact [9]. Therefore, this technique has also been used for white spot lesions (WSLs) on smooth tooth surfaces [10], as well as for enamel fluorosis [11, 12].

In ICDAS 2 and 3 lesions, there is still no distinct cavity, clinically speaking, even if signs of enamel defects are clearly visible. Radiographically, such caries lesions extend at most to the mid dentin [2]. In inactive lesions, the highly mineralized and mostly thick surface layer acts as a diffusion barrier against cariogenic acids; and, hence lesion progression is arrested, and RI is not indicated [13]. RI transforms active lesions into inactive ones [13] and it has been proven to be a safe and efficient method to arrest the progression of proximal initial caries lesions [14–16].

Acid-etching with strong acid (i.e., 15% hydrochloric acid) before the RI is mandatory to reveal the lesion under the demineralized outermost surface, and to enable penetration of resin into the porous caries lesion. By acid-etching with hydrochloric acid gel, resin penetration depths have been found to be higher than with phosphoric acid gel [17]. Interproximal lesions can be infiltrated with minimal tooth separation and with application strips that are coated on one side [18].

Experiences, attitudes, and manners regarding RI among pediatric dentists in the US were studied by means of an online survey by Halcomb et al. [19]. According to this study, the more education dentists had received on RI, the more often they would utilize it. Attitudes related to RI were positive and most of the respondents were eager to learn more about the technique. Of the participants, 28% used RI at least sometimes and 4% often or very often [19]. A questionnaire on teaching minimally invasive interventions in the undergraduate dental curriculum and pediatric dentistry specialty training programs was carried out in Iran [20]. All Iranian dental schools (40 undergraduate programs and 15 pediatric dentistry specialty training programs) participated in this study, and RI was considered to be one of the third least important preventive means among minimally invasive interventions. Half of the teachers in the undergraduate dental programs considered RI not essential in clinical training, whereas 25% of the respondents answered that RI would be important. On the other hand, among the directors in pediatric dentistry specialty training programs, 93% indicated that RI is essential [20].

In modern dentistry, noninvasive and tissue-saving methods are highly recommended. However, in Finland, invasive restorative caries procedures still account for nearly half of the dentists' working hours [21]. There is limited scientific evidence on the use of RI, possible barriers to its use, and ways to promote its use. The aim of this survey was to investigate the experiences, attitudes, and practices of RI among Finnish dentists.

## 2. Study Population and Methodology

### 2.1. Participants

A Webropol-based questionnaire was sent via the Finnish Dental Association's regular newsletter to members (dentists and dental students, *n* = 6687) in April 2022. The questionnaire was resent in the end of May 2022. The possibility to respond to the questionnaire ended in the middle of June 2022 with 176 responses. The same questionnaire was sent again in September 2022 to the superiors of the largest private dental companies in Finland, to the Finnish Student Health Services, and to the superiors in public health care to be delivered to all dentists. For bilingual areas the questionnaire was also sent in Swedish. A total of 416 responses were received in October 2022 when the questionnaire was finally closed.

### 2.2. Questionnaire

The following information was asked concerning background information of the respondents: The question on how often the respondents meet patients under 30 years of age had four answer options: daily/ weekly/less frequently than weekly/hardly ever. The university from which the respondents had graduated was also asked (Helsinki/Turku/Oulu/Kuopio) along with the number of years that had passed since their graduation (less than 5 years/5–10 years/more than 10 years).

The questionnaire had nine questions on dentists' experiences, utilization, and opinions of RI. For the question about the respondents' overall experience regarding the outcome of using RI, respondents could select one option from the following: positive/negative/I cannot say/I have not used it. For the question asking in what situations respondents had used RI, they were able to choose multiple options: initial caries lesions on interproximal surfaces of deciduous teeth/initial caries lesions in interproximal surfaces of permanent teeth/ molar incisor hypomineralization (MIH) teeth/WSLs of incisors/I have not used it. For the question on how the respondents had received information about RI, the options were: during undergraduate studies/ after graduate education/ introduced by a colleague/ from journals related to the field/ elsewhere, where? For this option there was a possibility to write a comment where the information was received and/I have not received any information.

For the question on opinions about RI, respondents could choose from several options: RI is a challenging procedure/RI is an easy procedure/ the scientific evidence of RI is promising/ according to my own experience, RI is promising in arresting caries lesion progression/ RI is promising in improving the esthetics of incisors with WSLs and arresting these lesions/ RI is a procedure that can also be performed by a dental hygienist /RI takes as much time as a filling/ RI requires (at least sometimes) local anesthesia/ RI is expensive/ no comment.

The methods that respondents had used for arresting initial caries lesions were also asked with a possibility to choose several options: I teach how to clean tooth surfaces with lesions/I prescribe a strong fluoride toothpaste for self-care/I apply fluoride varnish/gel on the initial lesions/I apply silver diamine fluoride (SDF) on the initial lesions/If necessary, I prepare individual fluoride trays for the patient/I seal the fissures of occlusal surfaces of children´s molars/I use RI/other, what? (Supporting Information).

### 2.3. Statistical Analyses

Descriptive analyses were performed using frequencies and proportions. Based on their answers to the question about their experience on the outcome of using RI, respondents were grouped into RI users and nonusers for the purpose of analysis. To analyze the association between the RI users and nonusers, cross tabulation with Pearson's chi-squared test or Fisher's exact test were used.

*p*-values <0.05 were considered statistically significant throughout the analyses.

Statistical analyses were executed with a statistical software package (SPSS for Windows Version 28.0.1.0 (142) IBM).

### 2.4. Ethical Considerations

Participation was voluntary and no compensation was given to the respondents. Anonymity was ensured by Webropol. The survey did not affect in physical or psychological integrity and according to Finnish legislation (488/1999), ethical approval of the Regional Medical Research Ethics Committee was not needed.

## 3. Results

Of the 416 respondents, one third (*n* = 136) answered that RI was included in their selection of procedures and 44 respondents were not interested in using it. More than 90% of the RI users treated children or young adults (under 30 years of age) at least weekly. The respondents included clinicians who had graduated from all four dental schools in Finland; furthermore responses were attained from 26 clinicians who had graduated outside of Finland. The majority of the RI users had graduated from the University of Oulu (40.4%) or the University of Helsinki (33.1%). More than half of the RI-users had graduated more than 10 years ago or earlier, but recent graduates (less than 5 years from graduation) also often had RI as part of their selection of procedures (Table 1).

Almost all respondents (*n* = 384) had received information about RI, and most frequently they received it during their undergraduate studies at the university (37%). The second common sources of information were further updating education courses (35%) and dental journals (35%). In the open-ended answers, the Internet and YouTube were mentioned most frequently. The most common indications for RI use were initial caries lesions on interproximal surfaces of permanent teeth (56%) and WSLs of incisors (54%). RI was also used on teeth with MIH by 15% of the respondents.

Of all the respondents, 90% responded that they instructed patients on better tooth brushing, prescribed strong fluoride toothpaste, or applied fluoride varnish/gel for arresting initial caries lesions. The prescribing of strong fluoride toothpaste was more common among RI users than nonusers (*p*=0.008). Sixty percent of the respondents reported sealing occlusal surfaces of permanent teeth. The use of individual fluoride trays was rare, but more common among RI users than nonusers (*p* < 0.001) (Table 2).

Almost half of the RI users considered RI an easy procedure, whereas one fifth of the nonusers had the same opinion. Scientific evidence of RI was reported to be promising by one third of the respondents, significantly more often by users than nonusers. Less than 10% of the nonusers considered RI promising for arresting initial caries lesions, whereas one fourth of the RI users held that belief. Compared to initial caries lesions, RI users considered the method more often to be promising when arresting WSL in incisors (42%).

Almost half of the RI users thought that RI could be performed by dental hygienists. RI procedures were considered time-consuming by one-fourth of the participants and expensive by one-fifth. The need for local anesthesia when performing RI was most seldom reported. Considering all the statements given, the differences in opinions between RI users and nonusers were statistically significant. One third had no opinion of the statements given (Table 3).

## 4. Discussion

Scientific evidence for the use of RI started only in the beginning of 2000s. Kugel et al. [22], shifted RI as a significant addition to the means to stop and arrest caries lesions progression. Today, RI seems to be one of the established caries prevention and arresting methods among Finnish clinicians who treat adolescents and young adults. According to this study, knowledge of RI has been received during undergraduate studies but also during continuing education and from dental journals. Perceptions of RI among all respondents and those who reported to be RI-users varied significantly. As a whole, RI users favored all caries preventive means more often than nonusers.

The Finnish Current Care Guidelines for clinical practice (caries [control]) [23] as well as ICCMS guidelines [2], recommend RI to arrest initial caries lesions. As expected and reported in previous studies [10], the main indications for RI in this study were initial interproximal caries lesions of the permanent teeth and WSL in permanent incisors, but also MIH lesions. Surprisingly, only one respondent had used RI in deciduous teeth. The reason might be the need for good cooperation from young children.

In this study, more than 70% of the RI-users had graduated from the Universities of Oulu or Helsinki. The explanation for this can be that RI was not adopted in the curriculums of the four Finnish Universities at the same time. This finding was in line with those reported from Iran, where the method was considered important by persons in-charge in universities but less frequently by dentists [20]. Interestingly, the use of RI was least frequent among those who had graduated five to 10 years ago and most frequent among most experienced clinicians. As more time passes since graduation, the basic measures are so well mastered that there is also more interest in embracing and learning new methods and procedures. Recent graduates have had the possibility to train in RI during their clinical studies with patients. Our study is partly in line with Halcomb et al. [19], in which dentists with higher education or more experience used RI more often. Networking and peer learning seem to be active, as colleagues have also been a source of information for a considerable proportion of RI-users.

Opinions regarding RI were different between RI-users and nonusers in the present study. RI-users appeared to be more interested in prevention methods, as they also mentioned prescribing strong fluoride toothpaste and prepare individual fluoride trays more frequently than the rest.

Today, a better understanding of dental caries as a complex and multifactored disease emphasizes minimal invasive dentistry instead of invasive restorative treatment [24]. It can be speculated that the vicious circle of “drilling and filling” can be avoided through the optimal use of a wide range of preventive means including RI. Here, the scientific evidence of RI was found to be promising significantly more often by RI-users than nonusers, especially in arresting WSL-lesions. An assumption of the degree of complexity can delay adoption of RI. According to the experiences of the RI-users in this study, the RI procedure requires local anesthesia and is time-consuming. Interestingly, a much larger proportion of RI-users than nonusers regarded RI as expensive. It has to be kept in mind that the costs of RI compared to ordinary fluoride products are high, but on the same level or even lower when compared to restorative treatments, not to mention the costs in the long run.

Controlling and arresting caries is an important part of dental hygienists' job responsibilities [25] and RI could be a good example of multiprofessional collaboration. In our study, RI-users in particular were of the opinion that RI could be seamlessly included in dental hygienists' scope of practise. Recently, expansion of the job description was also found to increase job motivation and grafting of hygienists in South Korea [26].

To our knowledge, this is the first study to evaluate this topic in Finland, which adds to its value. Despite our efforts and repeated possibilities for responding, the participation rate remained quite low, which is a shortcoming. Also, Halcomb et al. [19] encountered similar challenges in their study on pediatric dentists' experiences and attitudes regarding RI. However, the participation rate was in line with other recent questionnaire surveys carried out among dentists in Finland [27, 28]. To guarantee the anonymity of the respondents, we did not want to ask the specialty or the location of the respondents. Asking for more demographic facts of the respondents would have been interesting but is a topic in a future study. This survey did not include dental hygienists, who also can perform RI. The lack of their opinions and experience can also be considered as a limitation of the study. A positive aspect is that we received answers from dentists who had graduated from all four Finnish Universities and with varying amounts of work experience. In future, it would be interesting to repeat the survey and to find out if the perceptions and attitudes have changed. The experiences and opinions of dental hygienists would also be noteworthy to investigate.

## 5. Conclusion

Overall, the attitude of those who use RI in their clinical practice was more positive than that of the nonusers. This indicates a need for further promotion of this mini-invasive approach in caries treatment and RI as one important measure.

## 6. Clinical Relevance

RI is an appropriate additional tool in arresting the progression of initial caries lesions, and it is well in line with modern caries treatment schema. This mini-invasive method delays or even prevents the need for restorative treatment, especially of lesions on interproximal surfaces. Its ability to return the optical properties of affected enamel is an indication of its use for WSLs. According to the RI-users experience in this study, RI is also a procedure that can well be performed by a dental hygienist.

## Figures and Tables

**Table 1:** Descriptive statistics of the respondents (*n* = 416).

Variable	All respondents	The respondents whose selection of procedures included resin infiltration (RI)
	*n* = 416 (%)	*n* = 136 (32.7%)
Treating patients under 30 years of age		
Daily	255 (61.3)	86 (63.2)
Weekly	116 (27.9)	39 (28.7)
Less frequently than once a week	35 (8.4)	10 (7.4)
Very seldom or hardly ever	10 (2.4)	1 (0.7)
Graduation university		
Helsinki	121 (29.1)	45 (33.1)
Turku	75 (18.0)	14 (10.3)
Kuopio	54 (13.0)	14 (10.3)
Oulu	140 (33.7)	55 (40.4)
Elsewhere	26 (6.3)	8 (5.9)
Time from graduation		
Less than 5 years	132 (31.7)	47 (34.6)
5–10 years	73 (17.5)	25 (18.4)
Over 10 years	211 (50.7)	64 (47.1)

**Table 2:** Respondents' experiences related to resin infiltration (RI).

	All respondents *n* (%)	RI users *n* (%)	*p*-Value^a^
The source of information about RI			
During studies	154 (37.0)	61 (44.9)	0.021
Further education courses	146 (35.1)	55 (40.4)	n.s.
Colleagues	99 (23.8)	42 (30.9)	0.018
Dental journals	146 (35.1)	47 (34.6)	n.s.
Elsewhere	40 (9.6)	24 (17.6)	<0.001
Situations where respondents had used RI			
Initial caries lesions in proximal surfaces of deciduous teeth	—	1 (0.7)	—
Initial caries lesions in proximal surfaces of permanent teeth	—	76 (55.9)	—
MIH^b^ teeth	—	21 (15.4)	—
WSL^c^ of incisors	—	73 (53.7)	—
The methods that respondents have used for when arresting initial caries lesions (except RI)^d^			
Teaching how to clean tooth surfaces with initial lesions	380 (94.1)	125 (93.3)	n.s.
Prescribing strong fluoride toothpaste	363 (89.9)	128 (95.5)	0.008
Applying fluoride varnish/gel on the initial lesions	368 (91.1)	120 (89.6)	n.s.
Applying SDF^e^ to the initial lesions	39 (9.7)	18 (13.4)	n.s.
Preparing individual fluoride trays for the patient	14 (3.5)	11 (8.2)	<0.001
Sealing the occlusal surfaces	251 (62.1)	86 (64.2)	n.s.
Other	23 (5.7)	9 (6.7)	n.s.

^a^Chi-square or Fisher's exact test.

^b^Molar incisor hypomineralisation.

^c^White spot lesions.

^d^missing values *n* = 12.

^e^Silver diamine fluoride.

**Table 3:** Opinions regarding resin infiltration (RI) by users and nonusers of RI.

Statement	Agrees with statement *n* (%)			
	RI users *n* = 136	RI non-users (*n* = 280)	Total (*n* = 416)^a^	*p*-Value^b^
RI is an easy procedure	63 (46.7)	55 (19.9)	118 (28.6)	<0.001
Scientific evidence of RI is promising	56 (41.5)	78 (28.2)	134 (32.5)	0.007
RI is promising in arresting initial caries lesions	31 (23.0)	23 (8.3)	54 (13.1)	<0.001
RI is promising in arresting WSL^c^ lesions in incisors	57 (42.2)	20 (7.2)	77 (18.7)	<0.001
RI can also be performed by dental hygienist	65 (48.1)	45 (16.2)	110 (26.7)	<0.001
RI requires local anesthesia	18 (13.3)	10 (3.6)	28 (6.8)	<0.001
RI is as time consuming as filling	60 (44.4)	40 (14.4)	100 (24.3)	<0.001
RI is an expensive procedure	49 (36.3)	40 (14.4)	89 (21.6)	<0.001
No opinion	10 (7.4)	113 (40.8)	123 (29.9)	

^a^Missing answers total *n* = 4, 1 RI user 3 nonusers.

^b^Pearson chi-square test.

^c^White spot lesions.

## Data Availability

The data that supports the findings of this study can be requested from the authors.

## Supporting Information

Supporting Information English version of the questionnaire is attached as a supporting information file.
