# Supplementary material for: Experiences, Attitudes, and Practices of Resin Infiltration Use in Finland
Source: Int J Dent. 2025 Nov 19;2025:3400651. doi: 10.1155/ijod/3400651 (PMC12657073; doi:10.1155/ijod/3400651)
Supplement: Supporting Information — English version of the questionnaire is attached as a supporting information file. [file 3400651.f1.docx]

Questionnaire about resin infiltration

1. Is resin-infiltration included in your selection of procedures?
   1. yes, it is included if needed
   2. does not include but I am interested
   3. does not include and I am not interested
2. In what situation have you used resin-infiltration? (You can choose several options)
   1. initial caries lesions in interproximal surfaces of deciduous teeth
   2. initial caries lesions in interproximal surfaces of permanent teeth
   3. MIH teeth (molar-incisal hypoplasia)
   4. white spot lesions of incisors
   5. I have not used
3. Where have you got information about resin-infiltration? (You can choose several options)
   1. during undergraduate studies
   2. after graduate education
   3. introduced by a colleague
   4. from journals related to the field
   5. from elsewhere, where _________________
   6. I have not received any information
4. Which of these describe resin-infiltration in your opinion? (You can choose several options)
   1. RI is a challenging procedure
   2. RI is an easy procedure
   3. the scientific evidence of RI is promising
   4. according to my own experience, RI is promising in arresting caries lesion progression
   5. RI is promising in improving the aesthetics of incisors whit white spot lesions (WSL) and arresting these lesions
   6. RI is a procedure that can also be performed by a dental hygienist
   7. RI takes as much time as a filling
   8. RI requires (at least sometimes) local anesthesia
   9. RI is expensive
   10. no comment
5. My own experience about the outcome from using resin-infiltration are
   1. positive
   2. negative
   3. I cannot say
   4. I have not used
6. Methods that I have used for stopping the initial lesions (you can choose several options)
   1. I teach how to clean tooth surfaces with lesions
   2. I prescribe a strong fluoride toothpaste for self-care
   3. I apply fluoride varnish/gel on the initial lesions
   4. I apply silver diamine fluoride (SDF) to the initial lesions
   5. If necessary, I prepare individual fluoride trays for the patient
   6. I seal the fissures of occlusal surfaces of children’s molars
   7. I use RI
   8. other, what _________________________
7. In my reception visits under 30 years old patients
   1. daily
   2. weekly
   3. less frequently than once a week
   4. hardly ever
8. I have graduated from
   1. Helsinki
   2. Turku
   3. Kuopio
   4. Oulu
   5. somewhere else
9. How long has it been since you graduated?
   1. less than 5 years
   2. 5-10 years
   3. more than 10 years
10. Other comments
